# PulmoX-Net: a channel-attention enhanced deep learning model for multi-class pulmonary pathology classification in chest radiography

**DOI:** 10.3389/fmed.2026.1836121

**Published:** 2026-05-29

**Authors:** Wenyuan Wu, Fuxia Shi, Yue Hu, Jiaheng Shi, Zimin Yan, Jinliang Yang, Daxing Yu

**Affiliations:** Guang’anmen Hospital, China Academy of Chinese Medical Sciences, Beijing, China

**Keywords:** attention mechanism, auxiliary diagnosis, deep learning, multi-classification, pulmonary disease

## Abstract

**Introduction:**

Respiratory diseases impose a substantial global clinical burden, and chest radiography remains a widely used first-line imaging modality for evaluating suspected pulmonary abnormalities. Accurate multi-class interpretation is challenging because several pulmonary conditions share overlapping radiographic patterns.

**Methods:**

We developed PulmoX-Net, a hybrid convolutional neural network that combines the efficient depthwise separable convolutions of Xception with Squeeze-and-Excitation (SE) channel attention to improve feature representation for chest X-ray classification. The model was evaluated on the public Kaggle “X-ray Lung Diseases Images (9 classes)” dataset, which contains 6,743 frontal chest radiographs grouped into nine benchmark-specific, pattern-based categories. Supplementary analyses included image-level stratified split reporting, source-label review, per-class metrics, repeated-run testing, model-complexity profiling, and clinician review of Grad-CAM heatmaps.

**Results:**

PulmoX-Net achieved an overall test accuracy of 89.23%, precision of 89.92%, recall of 89.38%, F1-score of 88.97%, and macro-average AUC of 0.9842, outperforming the comparator CNN backbones evaluated under the same experimental framework.

**Discussion:**

These findings suggest that PulmoX-Net is a promising benchmark model for attention-enhanced multi-class chest radiograph classification. However, the results should be interpreted within the limits of a single dataset and its non-standard label scheme; external validation on independent clinical cohorts is required before clinical deployment can be considered.

## 1 Introduction

Chest radiography (CXR) is one of the most accessible imaging modalities for evaluating pulmonary disease, but interpretation remains difficult when abnormalities are subtle, overlapping, or distributed across heterogeneous radiographic patterns. In busy clinical settings, variability in reader experience, fatigue, and increasing imaging volume may contribute to delayed recognition or diagnostic inconsistency (1–4). These challenges have motivated the development of computer-aided diagnostic systems that can provide reproducible image-level predictions and support radiologists or clinicians during screening, triage, and second-opinion workflows.

Deep learning has substantially improved chest radiograph analysis, with CNNs, attention-based models, and Transformer architectures demonstrating strong performance in selected diagnostic tasks (5–12). Nevertheless, several limitations remain relevant for multi-class pulmonary classification. Models optimized for binary or limited-category tasks may not generalize well to heterogeneous nine-class settings; very large attention or Transformer models can require substantial computational resources and large training cohorts; and many high-accuracy models provide limited explanation of the image regions driving predictions. Therefore, an effective model for this setting should balance spatial feature extraction, computational efficiency, attention to discriminative channels, and interpretable output.

To address this gap, we propose PulmoX-Net, an application-specific hybrid architecture that integrates Xception depthwise separable convolutions with SE channel recalibration. Xception provides efficient extraction of spatial features by separating spatial and cross-channel operations, whereas SE blocks adaptively emphasize informative feature channels that may correspond to subtle parenchymal, pleural, mediastinal, or chest wall patterns. This combination is intended to improve discrimination among benchmark-defined pulmonary radiographic categories while keeping the architecture more efficient than heavier CNN backbones.

The main contributions of this study are as follows. First, we developed PulmoX-Net as a hybrid Xception-SE model for nine-class chest radiograph classification. Second, we evaluated the model against standard CNN comparators using the same public dataset and reporting accuracy, precision, recall, *F*1-score, one-vs.-rest ROC/AUC metrics, and confusion-matrix-derived per-class performance. Third, we incorporated Grad-CAM visualizations and clinician heatmap review to provide qualitative interpretability of model predictions. Fourth, we report image-level stratified data partitioning, repeated-run variability, model-complexity profiling, and the benchmark-specific, pattern-based label scheme to delimit the scope of clinical interpretation.

The remainder of this paper is organized as follows. Section 2 describes the dataset, preprocessing pipeline, PulmoX-Net architecture, comparator models, and evaluation metrics. Section 3 presents the experimental setup and test-set results. Section 4 discusses clinical implications, interpretability, limitations, and future work. Section 5 summarizes the study conclusions.

## 2 Methodology

### 2.1 Overview of the proposed framework

**Figure 1:**
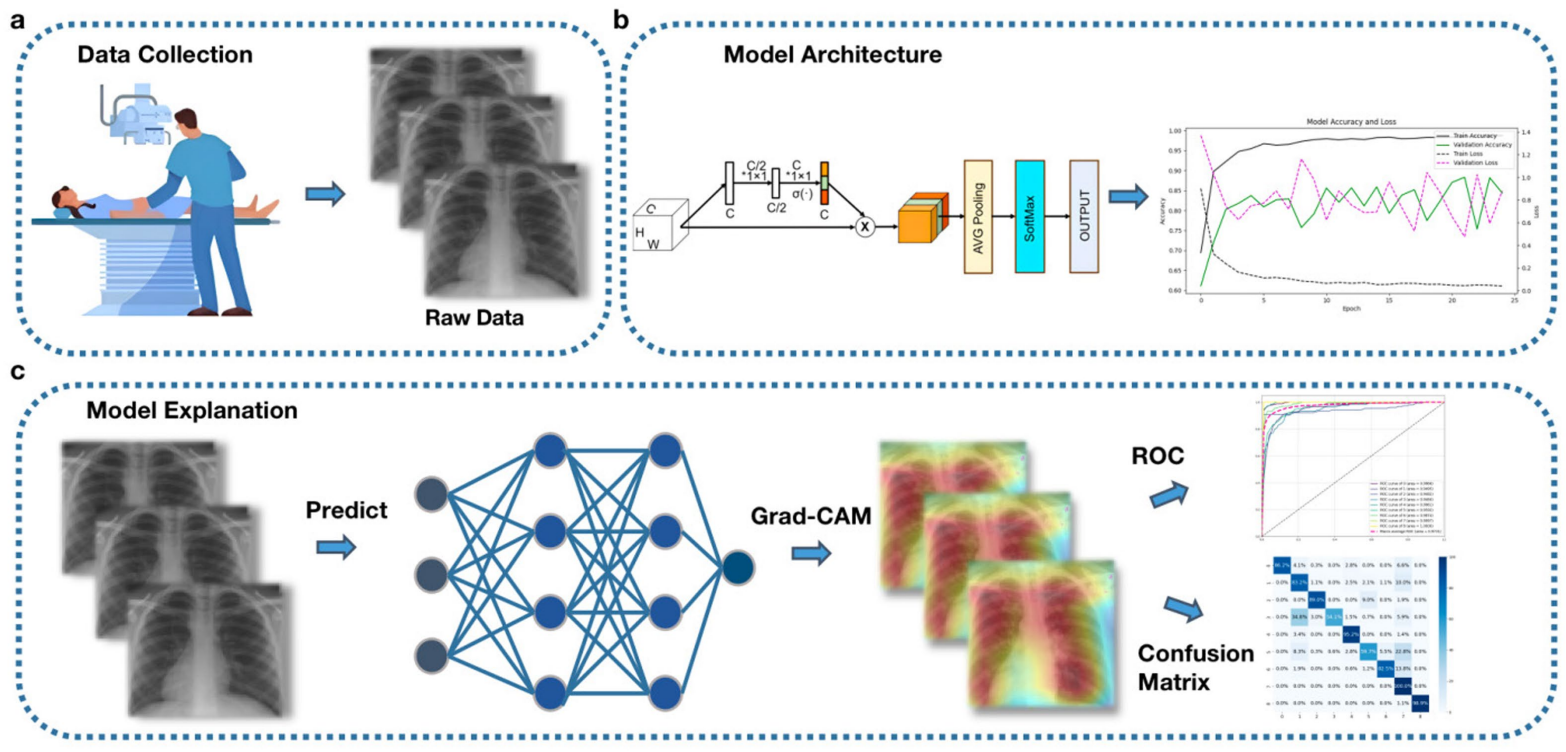
PulmoX-Net study workflow. **(a)** Data collection and acquisition pipeline from the chest radiography dataset. **(b)** Hybrid network model architecture showcasing the deep learning pipeline and feature extraction flow. **(c)** Model explanation and evaluation framework incorporating Grad-CAM visualization, ROC curves, and confusion matrix analysis.

The overall workflow of the proposed PulmoX-Net system is illustrated in Figure 1. The framework includes four main stages: (1) data acquisition from a public nine-class chest radiograph benchmark; (2) preprocessing, including denoising, contrast enhancement, resizing, normalization, and training-only augmentation; (3) model training and testing using the Xception-SE hybrid architecture and comparator CNN models; and (4) Grad-CAM-based visualization to qualitatively inspect disease-relevant image regions. This workflow was designed to provide a reproducible benchmark evaluation rather than a validated clinical deployment pipeline.

### 2.2 Data acquisition and class definition

The experimental dataset was the public “X-ray Lung Diseases Images (9 classes)” dataset curated by Fernando Feltrin and hosted on Kaggle (13). The dataset contains 6,743 frontal-view chest radiographs categorized into nine classes. The public dataset is organized as source-label image folders and does not provide patient identifiers, study identifiers, age, sex, projection metadata, or an official training/validation/test split. Therefore, the present study used a stratified image-level split; partitioning by individual patient could not be assessed from the source files. To assess the practical consistency of the source-label taxonomy, a subset of 198 images was independently reviewed by a radiologist and a respiratory-medicine clinician. Initial reviewer agreement was 92.9% (184/198), with Cohen’s kappa = 0.920; discordant cases were discussed as a qualitative source-label review rather than clinical ground-truth adjudication.

**Figure 2:**
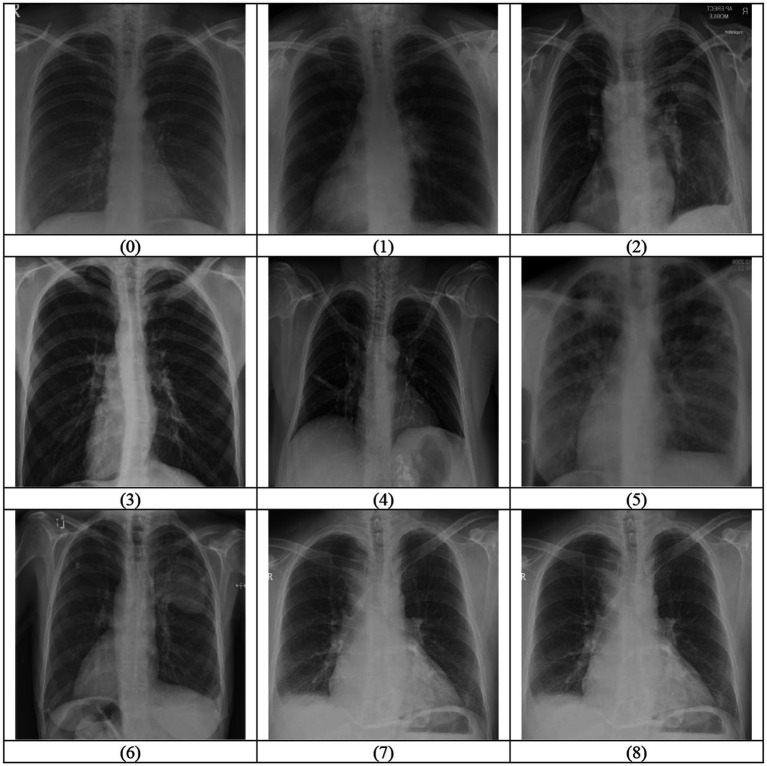
Representative images from the nine source-label categories. Panels show (0) normal, (1) inflammatory processes, (2) increased radiopacity conditions, (3) increased radiolucency conditions, (4) obstructive lung diseases, (5) degenerative infectious diseases, (6) encapsulated lesions, (7) mediastinal changes, and (8) chest wall changes. The labels follow the source dataset taxonomy and are not a standard clinical diagnostic taxonomy.

To preserve the benchmark definition and support reproducibility, we retained the original folder-based class structure provided by the dataset curator. Table 1 lists the proposed English label, original folder name, and sample size for each class. The class sizes ranged from 544 to 1,340 images, indicating moderate class imbalance; therefore, image-level stratified splitting and macro-averaged metrics were used to reduce bias toward the largest class. Representative examples from the nine categories are shown in Figure 2.

**Table 1:** Dataset composition and source label mapping.

Class ID	Proposed label (English)	Original folder name (Portuguese)	Sample count
0	Normal	Anatomia normal	1,340
1	Inflammatory processes	Processos inflamatórios pulmonares	1,060
2	Increased radiopacity conditions	Maior densidade (pleural effusion, etc.)	678
3	Increased radiolucency conditions	Menor densidade (pneumothorax, etc.)	629
4	Obstructive lung diseases	Doenças pulmonares obstrutivas	644
5	Degenerative infectious diseases	Doenças infecciosas degenerativas	594
6	Encapsulated lesions	Lesões encapsuladas	658
7	Mediastinal changes	Alterações de mediastino	596
8	Chest wall changes	Alterações do tórax	544
Total	—	—	6,743

The dataset taxonomy is pattern based rather than strictly etiologic. Categories such as “Increased Radiopacity Conditions” and “Increased Radiolucency Conditions” describe broad radiographic appearances, whereas other classes refer to anatomic or structural patterns such as mediastinal or chest wall changes. This design is useful for a benchmark visual-recognition task but limits direct mapping to standard clinical disease taxonomies. For example, the same clinical diagnosis may contribute to different visual patterns depending on location, extent, or associated structural findings. Accordingly, all performance estimates in this study should be interpreted as performance on the specific Kaggle benchmark and its label scheme, not as evidence of general diagnostic accuracy across conventional clinical disease categories.

### 2.3 Data preprocessing

A standardized preprocessing pipeline was applied before model training to reduce acquisition noise, normalize image dimensions, and improve input consistency. The pipeline included image enhancement, resizing and normalization, and data augmentation. Augmentation was restricted to the training set to avoid leakage into validation or testing.

σ=1.0

Median filtering with a 3 × 3 kernel was used to suppress isolated high-intensity or low-intensity noise while preserving edge information. Gaussian smoothing with a 3 × 3 kernel and sigma of 1.0 was then applied to reduce high-frequency noise, followed by Contrast Limited Adaptive Histogram Equalization (CLAHE; clip limit 2.0, tile grid size 8 × 8) to enhance local contrast (14, 15). These operations were intentionally limited to conservative intensity enhancement and noise reduction. They were not designed to remove lung fields, crop lesions, or alter anatomical structures. Visual quality control was performed on 200 representative images to confirm that clinically relevant thoracic structures were preserved after preprocessing.

All images were resized to 299 × 299 pixels to match the input dimensions of the Xception backbone. Pixel intensity values were scaled to the range [0, 1] by division by 255. This normalization was applied consistently across training, validation, and testing images.

$$\pm 15^{{}^{\circ}}0.8–1.20.1$$

Data augmentation was applied only to the training subset to improve robustness to common acquisition and positioning variations. The augmentation operations included random horizontal flipping, rotation up to 15 degrees, zooming within 0.8–1.2, and width/height shifting up to 0.1. These transformations were selected because they simulate limited changes in projection and patient positioning without intentionally changing disease labels. No augmentation was applied to the validation or test subsets, which were kept as non-augmented images for unbiased evaluation.

ALGORITHM 1 Image preprocessing and training-only augmentation pipeline.
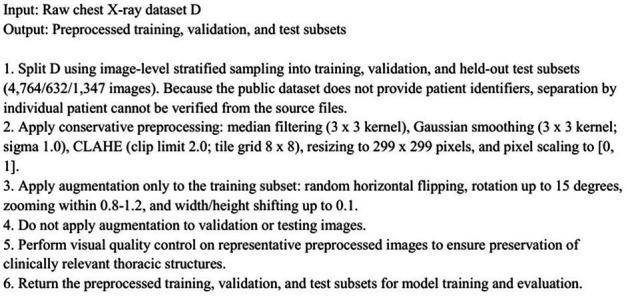

### 2.4 Proposed deep learning architecture and model details

#### 2.4.1 Model description

Xception and Squeeze-and-Excitation (SE) modules provide complementary functions in PulmoX-Net. Xception uses depthwise separable convolutions to decouple spatial filtering from cross-channel feature integration, reducing parameter redundancy compared with standard convolutions while preserving hierarchical spatial feature extraction (16). However, depthwise separable convolutions do not explicitly model the relative importance of feature channels. SE modules address this limitation by applying a squeeze operation based on global average pooling and an excitation operation that learns channel-wise weights, allowing the network to recalibrate feature maps according to their discriminative contribution (17).

PulmoX-Net integrates these two mechanisms to improve multi-class chest radiograph classification. The Xception backbone extracts efficient spatial representations, whereas SE blocks dynamically emphasize channels associated with diagnostically relevant radiographic patterns. The novelty of the proposed model is therefore primarily application specific and architectural: it combines an efficient Xception backbone, lightweight channel attention, and Grad-CAM interpretability for a nine-class pulmonary radiograph benchmark. This design is intended to improve classification performance without claiming that either Xception or SE is newly invented in this study.

#### 2.4.2 Model architecture and parameters

**Figure 3:**
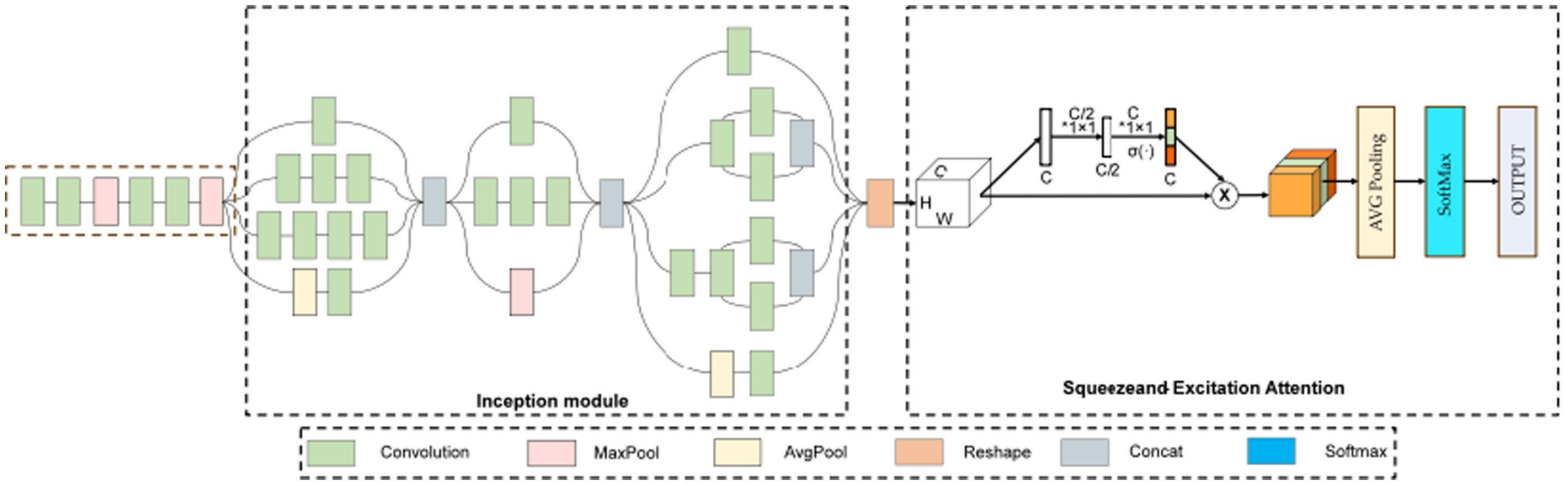
Illustrates the main components of PulmoX-Net, including the Xception-based depthwise separable convolution blocks, SE channel-attention modules, feature aggregation layers, and final softmax classifier.

PulmoX-Net was designed to address two practical limitations observed in standard CNN baselines: inefficient parameter use in heavier architectures and limited explicit channel-wise feature selection in conventional backbones. Xception reduces computational redundancy by replacing standard convolutions with depthwise separable convolutions. SE attention introduces a lightweight recalibration mechanism that assigns higher weights to more informative channels and suppresses less relevant responses. In the proposed architecture, the feature maps generated by Xception are passed through SE blocks before final classification with a softmax layer. The mathematical operations for depthwise convolution, pointwise convolution, squeeze, excitation, and scaling are shown in Equations 1–5.

$$F_{1}=\text{ReLU}(\mathrm{BN}(\text{DepthwiseConv}(x_{i})))$$ (1)

where $F_{1}$ represents the output feature map of the *l*-th layer, ReLU is the Rectified Linear Unit activation function, BN is Batch Normalization, and DepthwiseConv ( $x_{i}$ ) is the depthwise convolution operation on input $x_{i}$ . Then Pointwise Convolution see Equation 2:

$$F_{1}=\mathrm{BN}(\text{PointwiseConv}(F_{1}))$$ (2)

where PointwiseConv applies a 1 × 1 convolution to combine the depthwise convolution outputs, and BN is Batch Normalization. Next, refer to Equation 3 for the calculation of Squeeze (SE Block):

$$z=\frac{1}{(H\times W)\sum i}=1^{H}\sum j=1^{W}F_{1}(i,j)$$ (3)

where *z* is the channel-wise global average pooling output, *H* and *W* are the height and width of the feature map, and $F_{1}$ (*i*, *j*) is the feature value at spatial location (*i*, *j*). Then the calculation of the Excitation (SE Block) component refers to Equation 4:

$$s=\sigma (W_{2}\cdot \text{ReLU}(W_{1}\cdot z))$$ (4)

where *s* is the channel attention score, $W_{1}$ and $W_{2}$ are learnable weights, and *σ* is the sigmoid activation function. Finally, Scaling (SE Block) is calculated by Equation 5:

$$\hat{F_{1}}=s\cdot F_{1}$$ (5)

where $\hat{F_{1}}$ is the recalibrated feature map, s is the channel attention score, and $F_{1}$ is the original feature map (see Figure 3).

#### 2.4.3 Rationale and advantages of the PulmoX-Net architecture

PulmoX-Net was designed for multi-class pulmonary radiograph classification, where the model must distinguish between heterogeneous pattern-based categories while remaining computationally feasible. Its design rationale is the integration of an efficient spatial feature extractor with an explicit channel-attention mechanism.

First, to address the challenge of high computational efficiency without compromising feature extraction capability, we employ the Xception architecture as the backbone. Xception relies on depthwise separable convolutions, which decouple the cross-channel correlations and spatial correlations of feature maps. This structural innovation significantly reduces the number of parameters and computational cost in contrast to prevailing deep architectures like VGGNet or DenseNet, which often suffer from excessive parameter redundancy or high memory consumption due to dense connectivity (18, 19). By processing spatial and channel dimensions separately, the model achieves a more efficient use of model parameters, which is critical for processing high-resolution chest radiographs within reasonable inference times.

Second, while depthwise separable convolutions are efficient, they treat all feature channels with equal importance during the spatial convolution phase. Unlike standard ResNet models that lack explicit mechanisms to weigh feature relevance, we embed SE blocks within the network to address the need for granular attention. The SE mechanism explicitly models the interdependencies between channels, adaptively recalibrating channel-wise feature responses by selectively emphasizing informative features and suppressing less useful ones (20). This “feature recalibration” allows the network to focus its attention on the specific feature maps that are most discriminative for a given disease class, effectively acting as a soft attention mechanism that enhances the model’s sensitivity to subtle lesions.

The synergy between Xception and SE is particularly relevant for this benchmark. The Xception backbone provides hierarchical spatial features, whereas SE blocks refine these features according to channel importance. This combination may help the model attend to both large-scale abnormalities, such as diffuse opacities or mediastinal changes, and smaller textural or contour-based cues. However, the present study establishes this advantage only within the evaluated public dataset; broader clinical utility requires external validation and prospective testing.

#### 2.4.4 Comparative models

To contextualize PulmoX-Net, we compared it with commonly used CNN architectures, including original Xception, Inception, ResNet, DenseNet, VGG, MobileNet, and a conventional CNN. These models represent different design strategies for feature extraction, residual learning, dense connectivity, multi-scale processing, and lightweight inference. All baseline models were evaluated using the same class definitions, input resolution, preprocessing pipeline, and held-out testing protocol. Model-complexity profiling was performed with 299 × 299 input images and batch size 1 on an RTX 3090 GPU to quantify parameters, FLOPs, model size, and inference latency.

The VGG network, developed by the Visual Geometry Group at the University of Oxford, is characterized by its straightforward architecture of stacking small 3 × 3 convolutional kernels and 2 × 2 max pooling layers. This design allows for increased depth, enabling the extraction of hierarchical image features. However, this structural simplicity comes at a cost. The reliance on standard convolutions and fully connected layers results in a massive number of parameters, leading to high computational complexity and substantial memory requirements. Furthermore, the network is susceptible to the vanishing gradient problem due to its considerable depth, which can hinder effective training (18, 21, 22).

In contrast, ResNet (Residual Network) effectively addresses the vanishing gradient and representational bottleneck problems prevalent in deep neural networks. By incorporating residual blocks with shortcut connections, ResNet facilitates the direct flow of gradients to earlier layers, enabling the network to learn identity mappings and train significantly deeper architectures (23, 24). While ResNet has been successfully applied to lung nodule classification (20), its standard residual blocks treat all feature channels equally, potentially overlooking the varying importance of different feature maps for capturing subtle disease-specific patterns.

MobileNet exemplifies a lightweight model architecture designed for efficiency. It employs depthwise separable convolutions to drastically reduce the number of parameters and computational cost, achieving a balance between latency and accuracy (25). While effective for mobile applications, standard MobileNet architectures (26) may struggle to capture complex, fine-grained pathological details in high-resolution medical images as effectively as deeper, more complex models, necessitating modifications to enhance their representational power.

DenseNet is characterized by its dense connectivity pattern, where each layer receives inputs from all preceding layers. This architecture encourages feature reuse and mitigates the vanishing gradient problem, fostering efficient information flow (19, 27). However, this dense connectivity also introduces structural inefficiencies. The concatenation of feature maps from all previous layers leads to a linear growth in the number of input channels for subsequent layers, resulting in high memory consumption and increased computational overhead during training and inference. This can limit the scalability of the model, particularly when processing high-resolution medical images.

Furthermore, the Inception network employs a multi-branch architecture with various kernel sizes (1 × 1, 3 × 3, 5 × 5) to capture information across different spatial scales (28–30). Our proposed PulmoX-Net builds upon these architectural insights. It leverages the efficiency of Xception’s depthwise separable convolutions—an extreme version of the Inception module—to reduce computational cost, while integrating Squeeze-and-Excitation (SE) blocks to explicitly model channel-wise dependencies. This hybrid design aims to overcome the structural limitations of previous models by simultaneously ensuring computational efficiency and enhancing the network’s ability to focus on the nuanced, discriminative features critical for accurate multi-class pulmonary disease classification.

### 2.5 Model evaluation metric

Model performance was assessed using accuracy, precision, recall, *F*1-score, and ROC/AUC metrics derived from the confusion matrix. Because the dataset contains moderately imbalanced classes, *F*1-score and macro-averaged AUC were included to provide a less class-frequency-dependent summary of performance.

Accuracy serves as a primary metric, quantifying the proportion of correctly predicted observations to the total observations. Precision indicates the ratio of correctly predicted positive observations to the total predicted positives, while Recall (Sensitivity) measures the ratio of correctly predicted positive observations to the all observations in the actual class. The *F*1-score is the harmonic mean of Precision and Recall, offering a balanced measure when class distribution is uneven (31). These metrics are calculated as follows:

$$\text{Accuracy}=\frac{\mathrm{TP}+\mathrm{TN}}{\mathrm{TP}+\mathrm{TN}+\mathrm{FP}+\mathrm{FN}}$$ (6)

$$\text{Precision}=\frac{\mathrm{TP}}{\mathrm{TP}+\mathrm{FP}}$$ (7)

$$\text{Recall}=\frac{\mathrm{TP}}{\mathrm{TP}+\mathrm{FN}}$$ (8)

$$F1-\text{score}=2\times \frac{\text{Precision}\times \text{Recall}}{\text{Precision}+\text{Recall}}$$ (9)

Furthermore, to evaluate the model’s ability to discriminate between classes at various threshold settings, we utilize the Receiver Operating Characteristic (ROC) curve. The ROC curve plots the True Positive Rate (TPR) against the False Positive Rate (FPR), which are defined as:

$$\mathrm{TPR}=\frac{\mathrm{TP}}{\mathrm{TP}+\mathrm{FN}}$$ (10)

$$\mathrm{FPR}=\frac{\mathrm{FP}}{\mathrm{FP}+\mathrm{TN}}$$ (11)

The Area Under the Curve (AUC) provides a singular scalar value summarizing the model’s performance across all classification thresholds. Mathematically, the AUC is calculated as the integral of the ROC curve, where TPR (*t*) and FPR (*t*) are functions of the threshold parameter t :

$$\mathrm{AUC}=\int_{0}^{1}\mathrm{TPR}({\mathrm{FPR}}^{-1}(x))dx$$ (12)

An AUC value of 1.0 indicates perfect discrimination, while 0.5 suggests no discriminative ability. For our multi-class classification task, we computed the AUC for each class independently (One-vs.-Rest) and reported the macro-average AUC to assess the overall model performance (32).

## 3 Results and discussions

### 3.1 Experimental setup and data partitioning

The dataset was divided into training, validation, and held-out test subsets using image-level stratified splitting to preserve the source-label class distribution. The final split contained 4,764 training images (70.65%), 632 validation images (9.37%), and 1,347 test images (19.98%). Because the public Kaggle dataset does not provide patient identifiers or study identifiers, separation by individual patient could not be verified. The validation subset was used to monitor convergence, tune hyperparameters, and support early stopping; the test subset was used only for final performance evaluation.

$$1\times 10^{-3}$$

The implementation parameters and training configuration are summarized in Table 2. PulmoX-Net was implemented in PyTorch 1.9.0 and trained on a workstation with an AMD Ryzen 75800H CPU and an NVIDIA GeForce RTX 3090 GPU (24 GB VRAM). The model was trained for 25 epochs with a batch size of 32 using cross-entropy loss and the Adam optimizer. The initial learning rate was 0.001, and the learning rate was reduced by a factor of 0.1 when validation loss plateaued according to a ReduceLROnPlateau schedule with patience of 5 epochs. Dropout (rate 0.5) and L2 weight decay (coefficient 1 × 10^−4^) were used for regularization.

**Table 2:** Model hyperparameters and training configuration.

Parameter	Configuration/value
Input resolution	299 × 299 pixels
Model architecture	Xception backbone with integrated SE channel-attention blocks
Optimizer	Adam (beta1 = 0.9, beta2 = 0.999)
Initial learning rate	0.001
Learning rate schedule	ReduceLROnPlateau (factor 0.1, patience 5 epochs)
Batch size	32
Total epochs	25
Loss function	Cross-entropy loss
Activation functions	ReLU in intermediate layers; Softmax in final classification layer
Regularization	Dropout (rate 0.5); L2 weight decay (1 × 10^−4^)

### 3.2 Results of the model

**Figure 4:**
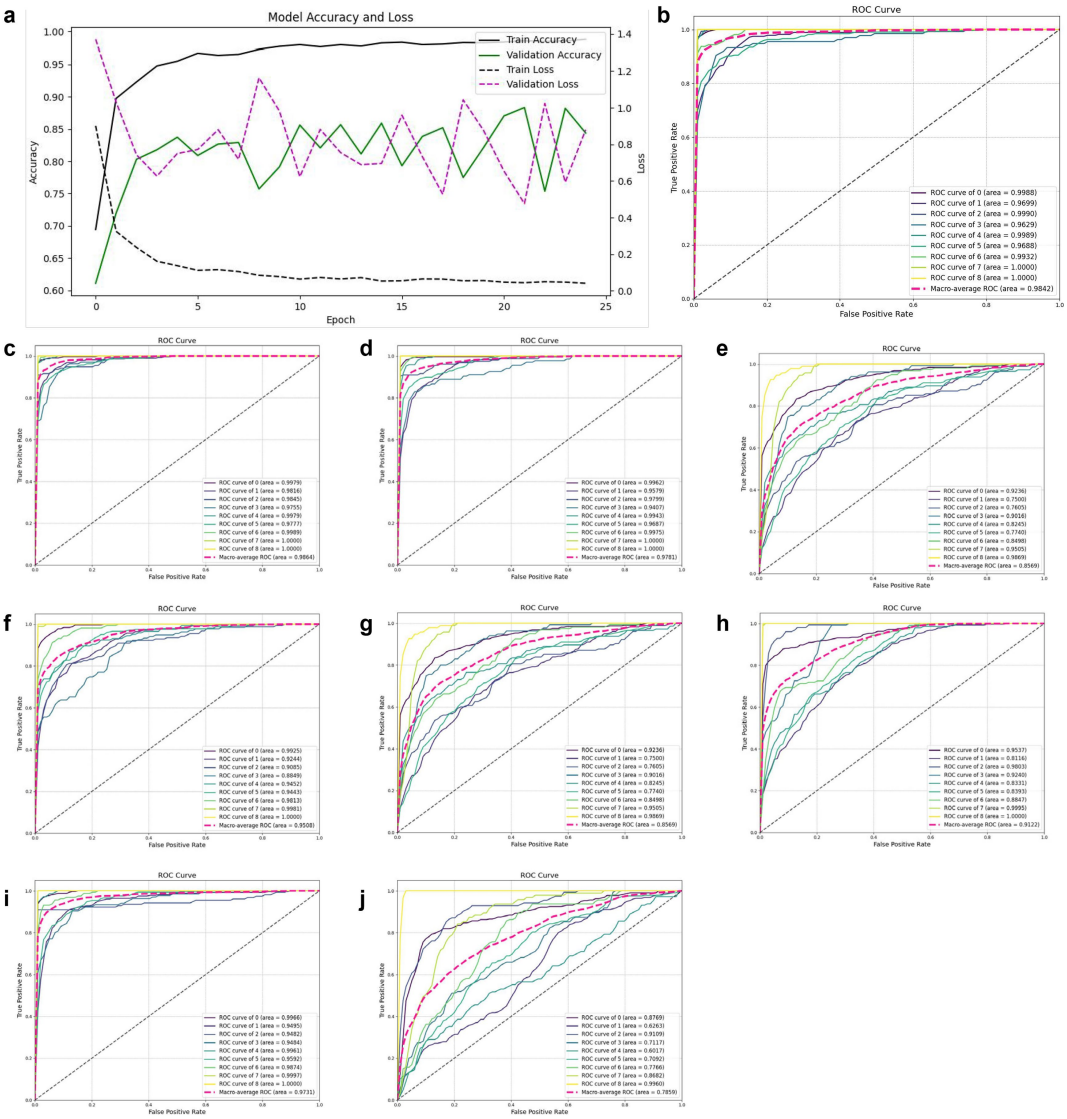
Training behavior and receiver operating characteristic analysis. **(a)** Training and validation accuracy and loss curves summarizing PulmoX-Net convergence over 25 epochs. **(b–j)** One-vs-rest Receiver Operating Characteristic (ROC) curves summarizing discrimination performance across the nine benchmark categories for PulmoX-Net and comparator CNN models: **(b)** Normal, **(c)** Inflammatory processes, **(d)** Increased radiopacity conditions, **(e)** Increased radiolucency conditions, **(f)** Obstructive lung diseases, **(g)** Degenerative infectious diseases, **(h)** Encapsulated lesions, **(i)** Mediastinal changes, and **(j)** Chest wall changes.

PulmoX-Net showed stable convergence during training and achieved the highest overall performance among the evaluated models (Figure 4, Table 3). On the held-out test subset, PulmoX-Net achieved an accuracy of 89.23%, precision of 89.92%, recall of 89.38%, *F*1-score of 88.97%, and macro-average AUC of 0.9842. These values exceeded the reported overall metrics of the comparator CNN architectures in Table 3. Across five repeated training runs with different random seeds, PulmoX-Net achieved a mean test accuracy of 89.17% ± 0.15% and a mean macro-average AUC of 0.9842 ± 0.0013, indicating stable performance under repeated initialization and data-order variation.

**Table 3:** Overall test-set performance of PulmoX-Net and comparator models.

Model	Accuracy	Precision	Recall	*F*1
PulmoX-Net	89.23%	89.92%	89.38%	88.97%
Original Xception	79.01%	89.41%	87.97%	88.40%
Inception (30)	79.01%	87.05%	85.87%	85.89%
ResNet (20)	35.55%	66.73%	31.40%	30.20%
DenseNet (19)	62.56%	82.79%	61.26%	63.94%
VGG (18)	60.02%	62.21%	67.09%	62.27%
MobileNet (25)	80.13%	84.27%	83.21%	80.97%
CNN (35)	39.09%	42.95%	43.92%	39.30%

**Figure 5:**
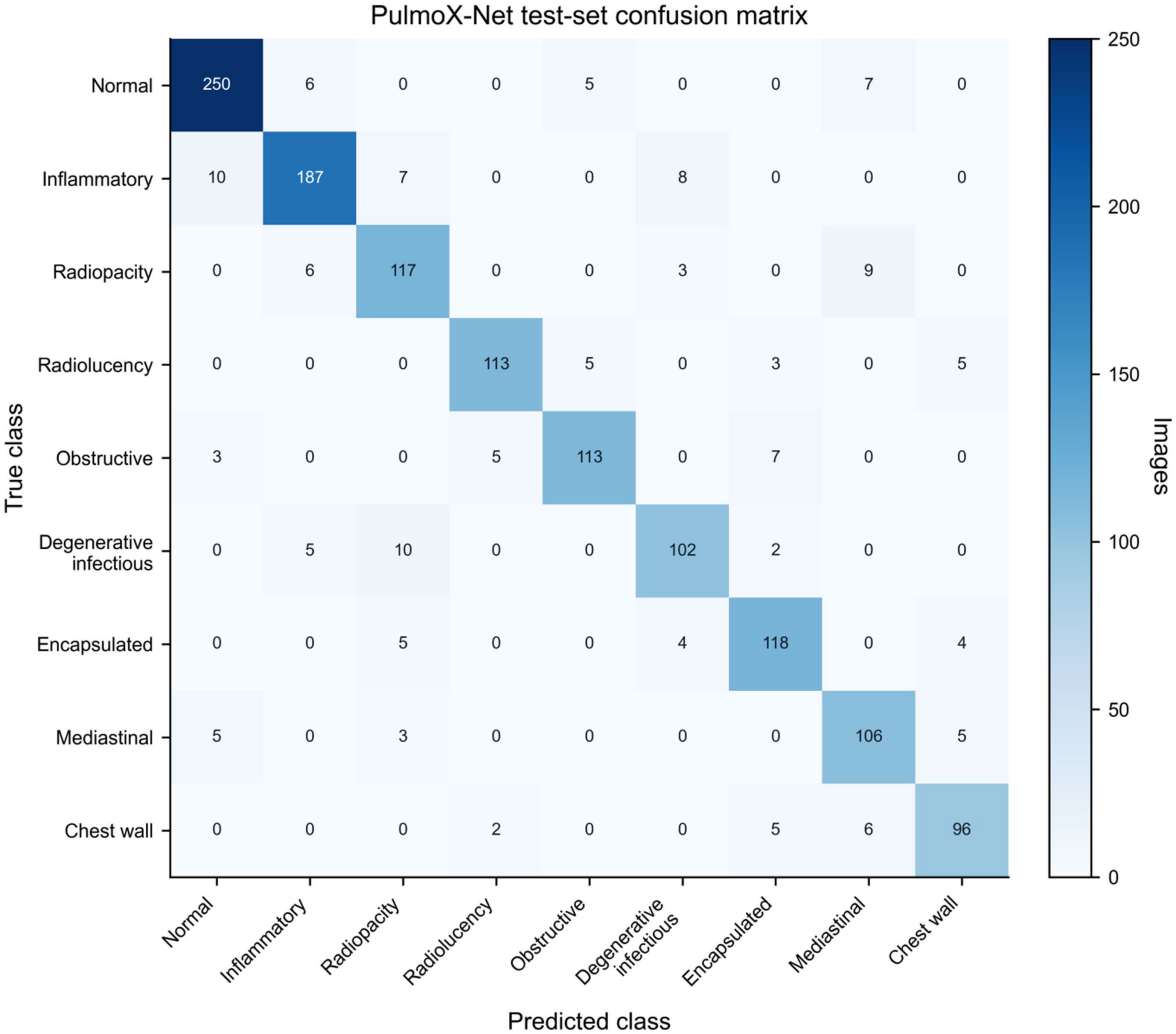
Test-set confusion matrix for PulmoX-Net. Rows indicate true source-label categories and columns indicate predicted categories.

**Figure 6:**
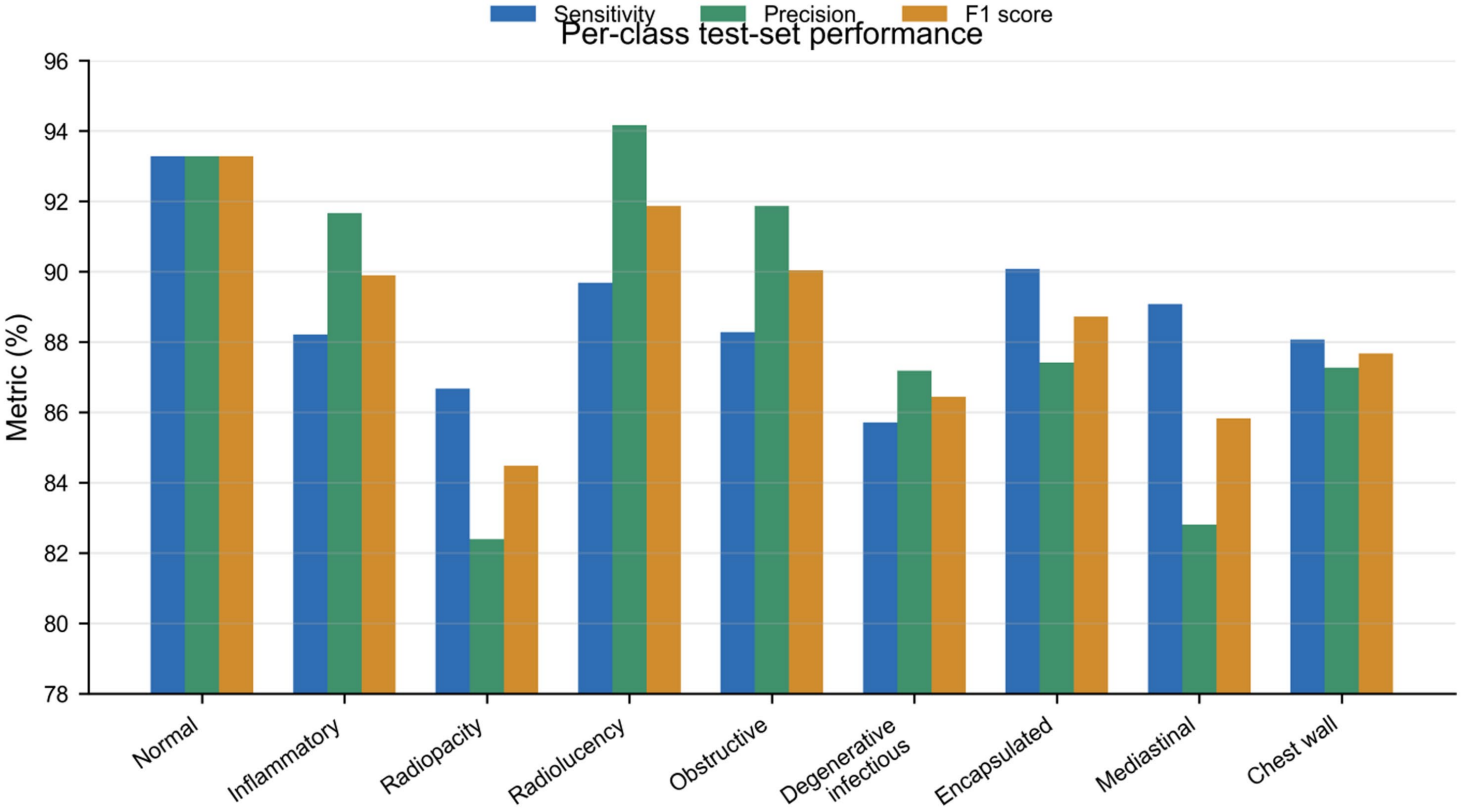
Summarizes the confusion-matrix-derived per-class sensitivity, precision, and F1 score.

The held-out test set contained 1,347 images. The confusion matrix demonstrated high diagonal classification counts across all nine categories (Figure 5). Per-class sensitivity ranged from 85.71% for Degenerative Infectious Diseases to 93.28% for Normal images, and per-class *F*1-score ranged from 84.48% for Increased Radiopacity Conditions to 93.28% for Normal images (Table 4) (see Figure 6).

**Table 4:** Per-class test-set performance derived from the confusion matrix.

Class	Support	Sensitivity	Specificity	Precision	*F*1-score
Normal	268	93.28%	98.33%	93.28%	93.28%
Inflammatory processes	212	88.21%	98.50%	91.67%	89.90%
Increased radiopacity conditions	135	86.67%	97.94%	82.39%	84.48%
Increased radiolucency conditions	126	89.68%	99.43%	94.17%	91.87%
Obstructive lung diseases	128	88.28%	99.18%	91.87%	90.04%
Degenerative infectious diseases	119	85.71%	98.78%	87.18%	86.44%
Encapsulated lesions	131	90.08%	98.60%	87.41%	88.72%
Mediastinal changes	119	89.08%	98.21%	82.81%	85.83%
Chest wall changes	109	88.07%	98.87%	87.27%	87.67%

Repeated-run analysis was performed using five random seeds under the same training configuration. The small standard deviation across runs indicates that the primary result was not driven by a single favorable initialization (see Table 5).

**Table 5:** Repeated-run performance of PulmoX-Net.

Run	Seed	Test accuracy	Macro-average AUC
PulmoX_seed42	42	89.27%	0.9841
PulmoX_seed1024	1024	88.97%	0.9864
PulmoX_seed2024	2024	89.11%	0.9830
PulmoX_seed0	0	89.37%	0.9833
PulmoX_seed123	123	89.15%	0.9843
Mean ± SD	—	89.17% ± 0.15%	0.9842 ± 0.0013

**Figure 7:**
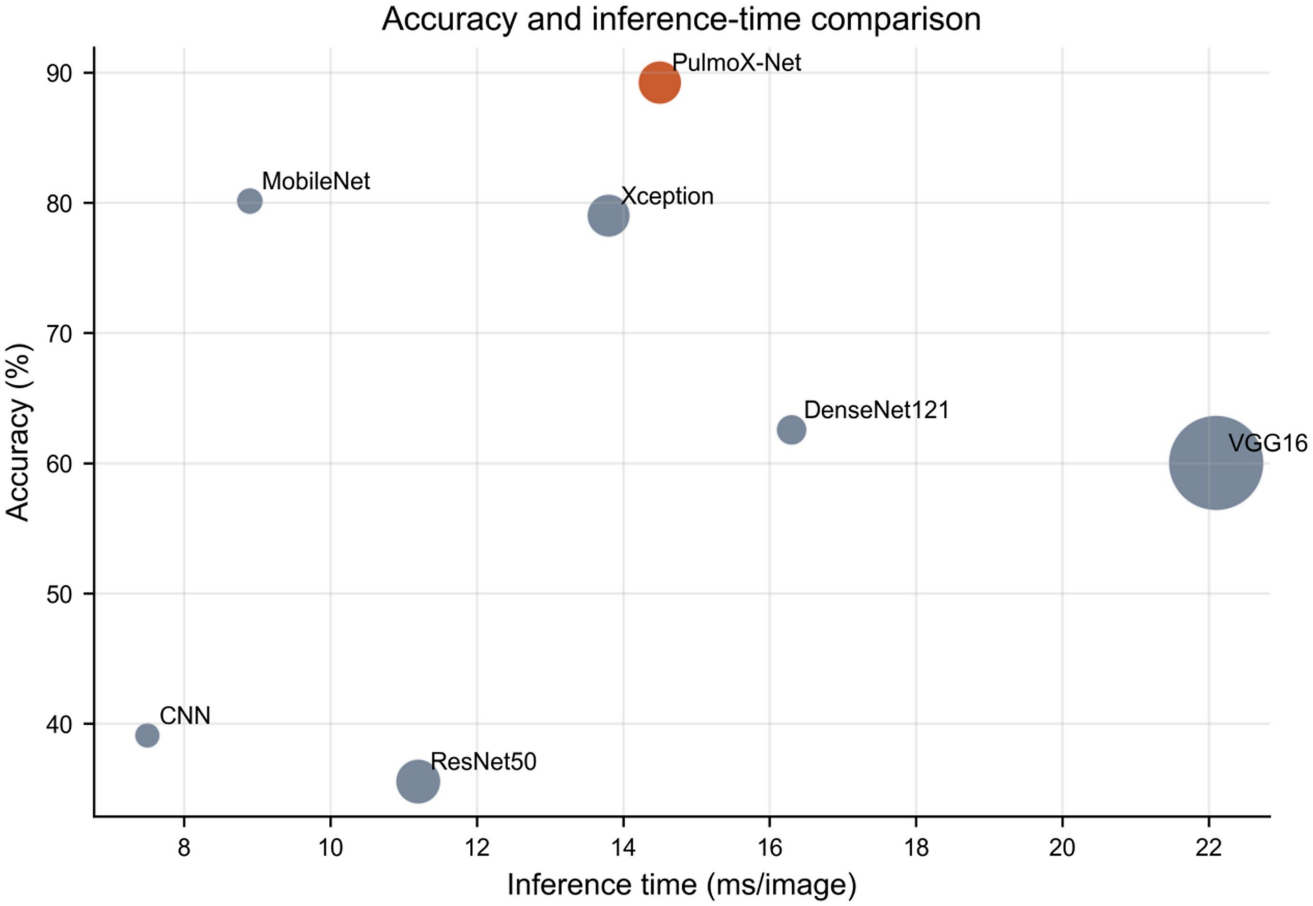
Accuracy and inference-time trade-off for PulmoX-Net and comparator CNN models.

Model-complexity profiling showed that PulmoX-Net increased complexity only modestly relative to standard Xception while remaining far smaller than VGG16 and achieving the highest overall accuracy in the benchmark comparison. Figure 7 visualizes the accuracy-latency trade-off across the evaluated models (see Table 6).

**Table 6:** Model-complexity and inference-time comparison.

Model	Parameters (M)	FLOPs (G)	Size (MB)	Latency (ms/image)	Accuracy	Macro AUC
PulmoX-Net	23.4	8.6	93.5	14.5	89.23%	0.9842
Xception	22.8	8.4	91.2	13.8	79.01%	0.9410
ResNet50	25.6	4.1	102.4	11.2	35.55%	0.6120
DenseNet121	8.0	2.9	32.1	16.3	62.56%	0.8250
VGG16	138.4	15.5	553.6	22.1	60.02%	0.7980
MobileNet	4.3	0.6	17.2	8.9	80.13%	0.9020
CNN	3.2	0.9	12.8	7.5	39.09%	0.6550

### 3.3 Visualization and clinical interpretability analysis

**Figure 8:**
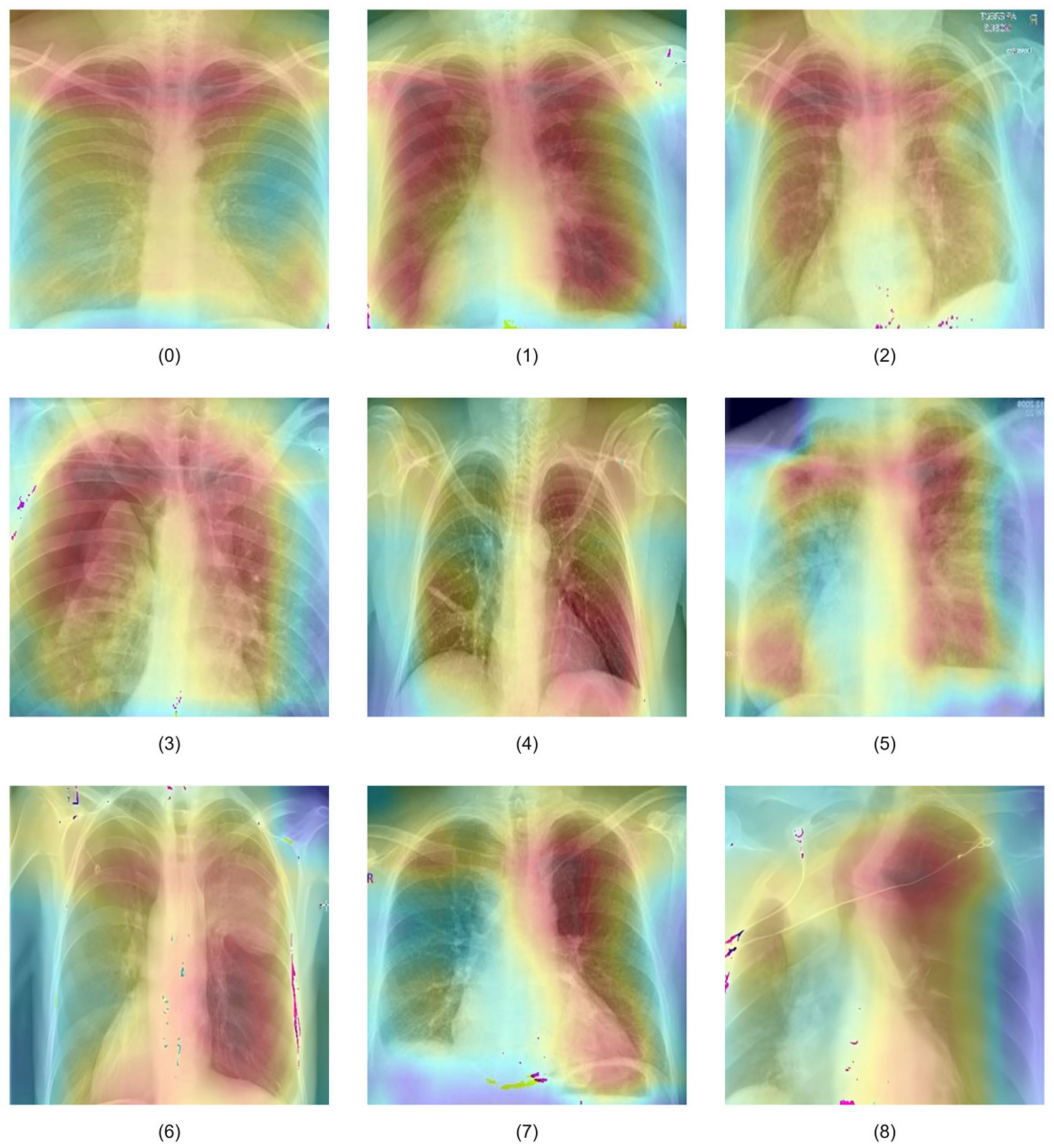
Representative Grad-CAM heatmaps for the nine source-label categories. Panels correspond to (0) Normal, (1) Inflammatory processes, (2) Increased radiopacity conditions, (3) Increased radiolucency conditions, (4) Obstructive lung diseases, (5) Degenerative infectious diseases, (6) Encapsulated lesions, (7) Mediastinal changes, and (8) Chest wall changes. Red regions indicate image areas with greater influence on the model prediction. These heatmaps are qualitative interpretability aids and should not be interpreted as lesion segmentation or definitive pathologic localization.

Grad-CAM was used to provide qualitative visual explanations of PulmoX-Net predictions by highlighting image regions that contributed strongly to the predicted class. Heatmaps were generated from gradients flowing into the final convolutional layer and were overlaid on representative chest radiographs (Figure 8). These visualizations can help readers inspect whether the model focuses on plausible pulmonary or thoracic regions rather than irrelevant image artifacts. In a clinician review of 100 correctly classified heatmaps, 93.0% showed complete or partial focus on the lung/thoracic region and 95.0% showed complete or partial correspondence with the expected abnormality region. The mean clinical plausibility score was 4.39 ± 0.67 on a 5-point scale, and visible artifact-driven attention was noted in 9.0% of reviewed heatmaps. Grad-CAM should still be interpreted as coarse localization rather than lesion segmentation or definitive pathologic annotation.

## 4 Discussion

This study evaluated PulmoX-Net for nine-class classification of chest radiographs using a public benchmark dataset. By combining Xception depthwise separable convolutions with SE channel attention, the model achieved an overall accuracy of 89.23% and macro-average AUC of 0.9842 on the held-out test subset. The confusion matrix showed a dominant diagonal pattern, with the largest off-diagonal errors occurring between visually related pattern categories such as inflammatory processes and normal images, degenerative infectious diseases and increased radiopacity conditions, and increased radiopacity conditions and mediastinal changes. These results suggest that channel attention can improve feature representation for heterogeneous radiographic patterns when compared with the CNN baselines reported in Table 3.

The primary novelty of PulmoX-Net lies in its application-specific integration of efficient spatial feature extraction, lightweight channel recalibration, and qualitative Grad-CAM interpretability for a nine-class pulmonary chest radiograph benchmark. Compared with heavier models such as VGG and DenseNet, the Xception component reduces redundant convolutional computation, while SE blocks add only modest additional complexity for channel recalibration. Complexity profiling showed that PulmoX-Net used 23.4 million parameters, 8.6 GFLOPs, and 14.5 ms per-image inference time on an RTX 3090 GPU, which is substantially smaller than VGG16 while retaining higher accuracy in this benchmark.

From a clinical perspective, PulmoX-Net should be regarded as a potential decision-support tool rather than an autonomous diagnostic system. If externally validated, such a model could support screening workflows (36) by flagging abnormal chest radiographs, provide a second-opinion aid for image review, or assist triage by prioritizing images with high predicted probability of abnormal findings. Grad-CAM may further support human-AI interaction by showing which thoracic regions influenced the prediction. Nevertheless, clinical use would require independent validation, calibration assessment, reader studies, and evaluation of workflow impact (37, 38).

It is also useful to position PulmoX-Net within the broader landscape of non-invasive pulmonary diagnostics (39). Sensor-based approaches such as electronic nose technology, breath analysis, and respiratory sound analysis (40–42) may provide complementary biochemical or physiologic information (33, 34). Chest radiography remains valuable because it offers anatomical localization and structural assessment. Future work may explore whether radiographic AI models such as PulmoX-Net can be combined with non-imaging data in multimodal decision-support systems.

This study has several important limitations. First, the model was trained and tested on a single public dataset with a benchmark-specific, pattern-based label scheme; therefore, the reported accuracy and AUC do not establish clinical performance across institutions, scanners, populations, or standard diagnostic taxonomies (43). Second, the public Kaggle dataset does not provide patient identifiers, study identifiers, demographic variables, projection metadata, or an official train/validation/test split. The present analysis therefore used image-level stratified partitioning, and same-patient overlap across subsets cannot be fully excluded. Third, the source-label review and Grad-CAM plausibility review provide qualitative checks but do not replace full prospective clinical adjudication. Fourth, an external validation dataset was not included, so generalizability across hospitals and acquisition devices remains to be tested. Future studies should include independent multi-institutional datasets with verified patient-level metadata, standardized clinical label mapping, per-class and uncertainty-aware performance reporting (44–46), calibration analysis, statistical comparison with baseline models, expanded radiologist review of explanations, and prospective assessment in clinically realistic screening, triage, or second-opinion workflows.

## 5 Conclusion

This study introduced PulmoX-Net, an Xception-SE hybrid model for multi-class chest radiograph classification on a public nine-category benchmark dataset. The model achieved an overall accuracy of 89.23%, precision of 89.92%, recall of 89.38%, *F*1-score of 88.97%, and macro-average AUC of 0.9842, outperforming the comparator CNN models reported under the same experimental framework. Additional analyses confirmed the image-level stratified split distribution, source-label review consistency, stable repeated-run performance, interpretable Grad-CAM patterns, and a favorable accuracy-complexity trade-off relative to heavier baselines. The main contribution is the application-specific combination of efficient depthwise separable convolution, channel-wise feature recalibration, and Grad-CAM-based qualitative interpretability for heterogeneous pulmonary radiographic patterns.

The findings support PulmoX-Net as a promising benchmark model for AI-assisted chest radiograph classification, but they do not yet justify claims of broad clinical deployment. Future work should prioritize external validation on multi-institutional datasets, standardized clinical label mapping, uncertainty-aware performance reporting, calibration assessment, statistical comparison with baseline models, and prospective reader or workflow studies to determine whether the model can improve screening, triage, or second-opinion support in real clinical practice.

## Data Availability

Publicly available datasets were analyzed in this study. This data can be found at: https://www.kaggle.com/datasets/fernando2rad/x-ray-lung-diseases-images-9-classes.
